# Editorial: Acute respiratory distress syndrome: Lung protective strategy

**DOI:** 10.3389/fmed.2022.1121297

**Published:** 2023-01-12

**Authors:** Chun Pan, Domenico Luca Grieco, Jian-Xin Zhou

**Affiliations:** ^1^Department of Critical Care Medicine, Zhongda Hospital, School of Medicine, Southeast University, Nanjing, China; ^2^Department of Anesthesiology and Intensive Care Medicine, Fondazione Policlinico Universitario A. Gemelli IRCCS, Catholic University of the Sacred Heart, Rome, Italy; ^3^Department of Critical Care Medicine, Beijing Shijitan Hospital, Capital Medical University, Beijing, China

**Keywords:** VILI, mechanical ventilation, ARDS, PEEP, respiratory mechanics, ECMO

The acute respiratory distress syndrome (ARDS) is a severe acute hypoxemic respiratory failure caused by an inflammatory response involving alveolar epithelium (inflammatory edema) and capillary endothelial (microvascular thrombosis), which is induced by various etiologies (1). In the intensive care unit, mechanical ventilation, combined with auxiliary management, is one of the most important supportive therapies for ARDS patients (2). The traditional goal of mechanical ventilation is to maintain oxygenation and ensure carbon dioxide removal through adequate alveolar ventilation (3). However, in the last 3 decades, raising evidence indicates that mechanical ventilation may itself be harmful through the occurrence of ventilator-induced lung injury (VILI), which activates (or worsens) systemic inflammation and induces multi-organ failure. VILI is the leading cause of death in ARDS patients (4). Current evidence and clinical guidelines have shown that a lung-protective strategy including low tidal volume (< 6–8 ml/kg of predicted body weight), plateau (< 28–30 cmH_2_O) and driving pressure (< 14 cmH_2_O), and prone position for at least 12–16 h per day may alleviate VILI and improve clinical outcome (2). The respective effects of positive end-expiratory pressure and extra-corporeal membrane oxygenation (ECMO) or carbon dioxide removal are still under investigation (5, 6). In this Research Topic collection “Acute Respiratory Distress Syndrome: novel insights in strategies to enhance lung protection”, five related articles are published to provide further insights in this field.

Up to now, only the use of low tidal volume and prone position have been supported by high-level clinical evidence (7, 8). Studies in mechanically ventilated ARDS patients have shown that the population that most benefits from prone position are patients with moderate-to-severe ARDS, which was defined as a ratio of the partial pressure of arterial oxygen (PaO_2_) to the inspired oxygen fraction (FiO_2_) of less than 150 mm Hg, measured with a positive end-expiratory pressure (PEEP) of at least 5 cmH_2_O (8). After the outbreak of COVID-19 pneumonia in 2020, medical resources limitation became an issue worthy of attention. Several centers have tried to conduct prone position in non-intubated patients undergoing oxygen therapy, especially during high-flow nasal oxygen.

In our collection in the Journal, Huang et al. conducted a systematic review and meta-analysis to evaluate the efficacy and safety of awake prone position in COVID-19 patients. Among 1,686 patients from ten randomized controlled trials, awake prone position significantly reduced intubation requirement (risk ratio: 0.84; 95% confidence interval [CI]: 0.74–0.96, *P* = 0.007). However, the study also revealed poor adherence to awake prone position protocols in real clinical practice, which represents a call for the establishment of standardized procedures that can be applied worldwide.

Not all ARDS patients respond positively to prone position. Improvement of oxygenation is usually used as a marker for the response of the prone position. However, the improvement of oxygenation is not the sole parameter indicating less-injurious ventilation in the prone position. Also, the lack of improvement in oxygenation with prone position does not indicate that this is not beneficial. Wang Z. et al. performed a retrospective analysis in ARDS patients undergoing prone position to evaluate the association of ventilatory ratio, which is a surrogate of alveolar dead space, with ventilator weaning endpoints. Study results showed the ventilatory ratio decreased more significantly within 4 h after prone positioning in patients with successful weaning, and that ventilatory ratio-improvement induced by prone position was independently associated with liberation from mechanical ventilation on day 28. Prone position-induced improvement in ventilatory ratio may represent a tool to identify patients in whom prone position maximize its benefits.

ARDS is a syndrome caused by multiple etiologies, and heterogeneity is one of its most distinguishing features. Classification of patients, either by biological or clinical markers, is a research priority for ARDS patients. In this Research Topic, three articles focused on this domain.

Studies have shown that baseline mechanical ventilation parameters, such as respiratory system driving pressure and mechanical power, are associated with clinical outcomes in ARDS patients. In Hu et al. study, a secondary analysis was performed in 1,000 ARDS patients included in the NHLBI ARDS Network's Fluid and Catheter Treatment Trials (FACTT). The results showed that the 28-day mortality was significantly lower in patients with early negative fluid balance (15%) than those with persistent-positive fluid balance (50%), with the latter group requiring higher tidal volumes, driving pressure and mechanical power. The hazard ratio of 28-day mortality for time-varying static driving pressure and mechanical was significant lower in patients with negative fluid balance, but not in persistent-positive fluid balance patients. These results show that persistent-positive fluid balance was characterized by a higher intensity of mechanical ventilation: further research is needed to determine the eventual causal relationship between respiratory system mechanical properties and fluid management.

Cheng et al. retrospectively analyzed a cohort of immunocompromised ARDS patients undergoing ECMO. Study results suggested that immunocompromised status was not an independent risk factor for unsuccessful weaning from ECMO. Importantly, for patients with pulmonary hemorrhage and aspiration-related ARDS, ECMO may be beneficial as a bridge therapy.

In Wang Y. et al. study, the China Critical Care Sepsis Trial (CCCST) database was used, and clinical outcomes in various patients with ARDS were analyzed according to different etiologies. The study enrolled a total of 2,138 patients with ARDS, and found that hospital mortality in pulmonary ARDS is higher compared with extrapulmonary ARDS; however, if ARDS coexist with sepsis (multi-organ failure), mortality is increased. The study recommended that sepsis-induced pulmonary ARDS should attract more attention from ICU physicians and be cautiously treated.

ARDS is a heterogeneous diseases: future research on lung-protective strategies should focus on personalizing interventions based on patients' individual mechanical or inflammatory phenotype (7–10).

## Author contributions

All authors listed have made a substantial, direct, and intellectual contribution to the work and approved it for publication.
